# Amniotic Biologics for Knee Osteoarthritis: Promise, Evidence, and Unmet Needs—A Narrative Review

**DOI:** 10.3390/biomedicines14071572

**Published:** 2026-07-14

**Authors:** Kuan-Hao Chen, Wen-Tien Wu, Ru-Ping Lee, Ting-Kuo Yao, Cheng-Huan Peng, Wen-Yan Lin, Kuang-Ting Yeh

**Affiliations:** 1 School of Medicine, Tzu Chi University, Hualien 970374, Taiwan; 110311158@gms.tcu.edu.tw (K.-H.C.); timwu@tzuchi.com.tw (W.-T.W.); peng0913@tzuchi.com.tw (C.-H.P.); 2 Department of Medical Education, Hualien Tzu Chi Hospital, Buddhist Tzu Chi Medical Foundation, Hualien 970473, Taiwan; 3 Department of Orthopedics, Hualien Tzu Chi Hospital, Buddhist Tzu Chi Medical Foundation, Hualien 970473, Taiwan; tkyao0318@me.com; 4 Institute of Medical Sciences, Tzu Chi University, Hualien 970374, Taiwan; fish@gms.tcu.edu.tw; 5 Department of Orthopedics, Dalin Tzu Chi Hospital, Buddhist Tzu Chi Medical Foundation, Chiayi 622401, Taiwan; 6 Graduate Institute of Clinical Pharmacy, Tzu Chi University, Hualien 970374, Taiwan

**Keywords:** intra-articular injection, amniotic suspension allograft, micronized dehydrated human amnion/chorion membrane, mdHACM, regenerative medicine, biologic therapy, patient-reported outcomes, disease-modifying osteoarthritis drug, infrapatellar fat pad, viscosupplementation

## Abstract

**Background**: Knee osteoarthritis (KOA) is a leading cause of chronic pain and functional disability worldwide. Despite the widespread use of intra-articular (IA) corticosteroids and hyaluronic acid (HA), their long-term efficacy remains limited. Amniotic and chorionic membrane-derived biologics have emerged as promising alternatives owing to their anti-inflammatory, anti-fibrotic, and regenerative properties. **Methods**: This narrative review employed a structured, transparency-oriented literature search approach across PubMed/MEDLINE, EMBASE, Cochrane CENTRAL, and ClinicalTrials.gov (2015–2025). Studies evaluating intra-articular amniotic or chorionic membrane biologics in knee OA and reporting clinical outcomes were included. **Results**: Nine studies met the inclusion criteria, encompassing randomized controlled trials, prospective cohort studies, and retrospective analyses. Across studies, IA amniotic and chorionic membrane biologics demonstrated clinically meaningful improvements in pain scores and functional outcomes, with a favorable safety profile and minimal adverse events reported. **Conclusions**: Intra-articular amniotic and chorionic membrane biologics represent a promising therapeutic option for knee OA, with emerging evidence supporting their anti-inflammatory and potentially chondroprotective effects. Standardized product characterization, rigorous randomized controlled trials, and long-term follow-up studies are needed to establish their definitive clinical role.

## 1. Introduction

Knee osteoarthritis (OA) is one of the most prevalent and burdensome musculoskeletal conditions worldwide. It affects an estimated 365 million individuals globally and is a leading cause of chronic pain, functional disability, and reduced quality of life [1]. The pathophysiology of knee OA is multifactorial and is now recognized as a whole joint disease [2]. It involves progressive degradation of articular cartilage, subchondral bone remodeling, synovial inflammation, meniscal degeneration, and periarticular soft tissue changes collectively driven by a complex interplay of mechanical, biochemical, and cellular mechanisms. Notably, the infrapatellar fat pad (IFP)—the largest intra-articular adipose depot in the knee—actively contributes to OA pathobiology through secretion of pro-inflammatory adipokines (leptin, resistin, adiponectin), modulation of synovial inflammation, and IFP fibrosis, the extent of which correlates with OA severity and pain [3,4]. This disease disproportionately affects individuals aged > 50 years and is strongly associated with obesity, prior joint injury, and the female sex. Moreover, the global prevalence of knee OA is projected to increase substantially in parallel with the aging population and increasing rates of metabolic comorbidities. Thus, it poses an escalating burden on healthcare systems worldwide [5].

The current management of knee OA follows a stepwise approach. It begins with conservative measures such as weight reduction, physical therapy, and oral analgesics. Thereafter, it progresses to intra-articular (IA) pharmacologic interventions before surgical consideration. Among IA therapies, corticosteroid and hyaluronic acid (HA) viscosupplementation have been the mainstay of nonsurgical treatment. However, the clinical utility of these agents is being increasingly scrutinized. Corticosteroids provide rapid but short-lived pain relief, typically lasting four–eight weeks. In addition, their repeated administration is associated with accelerated cartilage volume loss and subchondral bone changes on quantitative MRI [6]. Although HA viscosupplementation is generally safe, it has demonstrated inconsistent efficacy across clinical trials. Several meta-analyses and systematic reviews have concluded that its clinical benefit over placebo may be minimal and of questionable clinical significance [7]. Furthermore, emerging morphological evidence has identified granulomatous synovitis, adipositis, and subchondral osteomyelitis as rare but clinically significant complications following repeated hyaluronan-based intra-articular injections, further underscoring the need for safer and more durable therapeutic alternatives [8]. Thus, platelet-rich plasma (PRP) has emerged as an alternative biological option. This therapeutic approach provides growth factor-mediated anabolic and anti-inflammatory effects. However, significant heterogeneity in preparation protocols, platelet concentrations, and leukocyte content has precluded definitive conclusions regarding its clinical superiority over conventional IA comparators [9]. Similarly, stromal vascular fraction (SVF) derived from adipose tissue has been explored as a cellular biologic for OA, with emerging evidence suggesting structural benefits in the osteochondral unit; however, its clinical translation remains limited by procedural complexity and regulatory considerations [10]. Collectively, these limitations underscore the urgent need for novel IA biologics that can provide durable symptomatic relief while potentially exerting disease-modifying effects on the underlying pathobiological process—an unmet clinical need that amniotic and chorionic membrane biologics are uniquely positioned to address.

Human amnion and chorion membranes, the innermost layers of the placenta, have long been utilized in ophthalmic surgery, wound care, and reconstructive applications because of their unique biological properties, including anti-inflammatory, anti-fibrotic, and pro-regenerative activities [11]. These membranes are rich in various bioactive molecules, including epidermal growth factor, fibroblast growth factor, transforming growth factor-beta (TGF-β), platelet-derived growth factor, tissue inhibitors of metalloproteinases (TIMPs), and heavy chain-hyaluronic acid/pentraxin 3 complexes. Collectively, these biomolecules modulate inflammatory cascades, inhibit matrix metalloproteinase-mediated cartilage degradation, and support IA tissue homeostasis—mechanisms directly relevant to OA pathobiology [12,13]. The development of micronized dehydrated human amnion/chorion membrane (mdHACM) through the proprietary PURION^®^ processing technology has enabled the preservation of these bioactive constituents in a shelf-stable, devitalized allograft format amenable to point-of-care clinical application, including minimally invasive IA injection [14]. The clinical translation of this injectable formulation was first explored by Alden et al. [15], who reported significant improvements in KOOS Pain and functional outcomes at 6 months following a single 100 mg IA injection of mdHACM in a retrospective case series comprising 100 knees. These findings provided the initial human clinical signal that supported further investigation [15]. Subsequent preclinical investigations have sought to elucidate the mechanisms underlying the observed clinical effects.

Preclinical investigations have provided an early mechanistic rationale for the IA application of mdHACM in OA. Willett et al. [14] were the first to demonstrate that a single IA injection of mdHACM attenuated cartilage degradation and reduced proteoglycan loss in a rat medial meniscal transection model of OA. Their findings provided a foundational proof-of-concept for the disease-modifying potential of IA mdHACM and established that the material is rapidly sequestered within the synovial membrane following injection, potentially enabling sustained local bioactivity [14]. Salazar-Noratto et al. [16] further characterized the localized OA disease-modifying changes induced by mdHACM in preclinical models, suggesting that its bioactive payload can favorably alter the IA microenvironment. More recently, IA delivery of mdHACM reduced degenerative changes in a rat model of post-traumatic OA (PTOA), even when administered after disease onset [17]. These results have broadened the potential therapeutic window and suggest clinical applicability even in established disease states.

The translation of these preclinical findings into clinical practice began with human studies that explored the use of cryopreserved and dehydrated amniotic suspension products for knee OA. Vines et al. [18] were among the first to introduce the concept of a cryopreserved amniotic suspension for IA use, establishing its early feasibility and safety as a matrix-modulatory placental allograft in a clinical setting. The subsequent development of amniotic suspension allograft (ASA) formulations led to the first robust multicenter randomized controlled trial (RCT) by Farr et al. [19]. This 200-patient multicenter cohort demonstrated the statistically significant superiority of ASA over both HA viscosupplementation and saline placebo in KOOS Pain and symptom scores at 6 months [19]. The 12-month follow-up data [20] confirmed the durability of these effects. Gomoll et al. [20] reported sustained KOOS Pain improvement and a reassuring immunological profile, including the absence of clinically relevant anti-HLA antibody formation. More recently, Pill et al. [21] conducted a double-blind RCT of morselized amniotic tissue (BioDRestore) versus triamcinolone acetonide in patients with severe KL grade 3–4 knee OA. Their findings demonstrated short-term efficacy comparable with that of corticosteroid injections along a favorable trajectory of continued functional improvement extending through 12 months, a pattern not observed in the corticosteroid arm [21]. Despite this evolving evidence, direct prospective clinical data specifically evaluating mdHACM as an IA therapy for knee OA remain limited in both quantity and methodological rigor. The most substantive human evidence to date is the retrospective case series by Alden et al. [15]. Although this study demonstrated promising functional improvements, it was constrained by its uncontrolled design, single-arm retrospective methodology, and the absence of KL grade stratification. These limitations preclude definitive conclusions regarding efficacy. To date, no prospective, randomized, or concurrently controlled trials of mdHACM versus standard-of-care comparators have been published. Furthermore, critical evidence domains, including quantitative MRI-based structural outcomes, synovial and serum biomarker analyses, and formal cost-effectiveness evaluations, remain entirely unaddressed in the published clinical literature, representing critical lacunae that must be resolved before mdHACM can be considered for broader clinical adoption.

Given these substantial evidence gaps, a comprehensive and critical synthesis of the existing literature is required to guide clinicians, researchers, and regulatory stakeholders. The present narrative review, employing a structured, transparency-oriented search approach, aimed to: (1) comprehensively summarize the available clinical evidence for IA amniotic and chorionic membrane-derived biologics in knee OA; (2) characterize the safety and immunological profiles of these products across human studies; (3) identify and contextualize critical gaps in the current evidence base; and (4) propose a structured framework for future definitive clinical trials. By synthesizing evidence from both directly relevant mdHACM studies and closely related amniotic/chorionic membrane formulations, this review seeks to provide a holistic and clinically actionable perspective on the current state and future trajectory of IA amniotic and chorionic membrane biologic therapy for knee osteoarthritis.

## 2. Search Strategy

**Figure 1:**
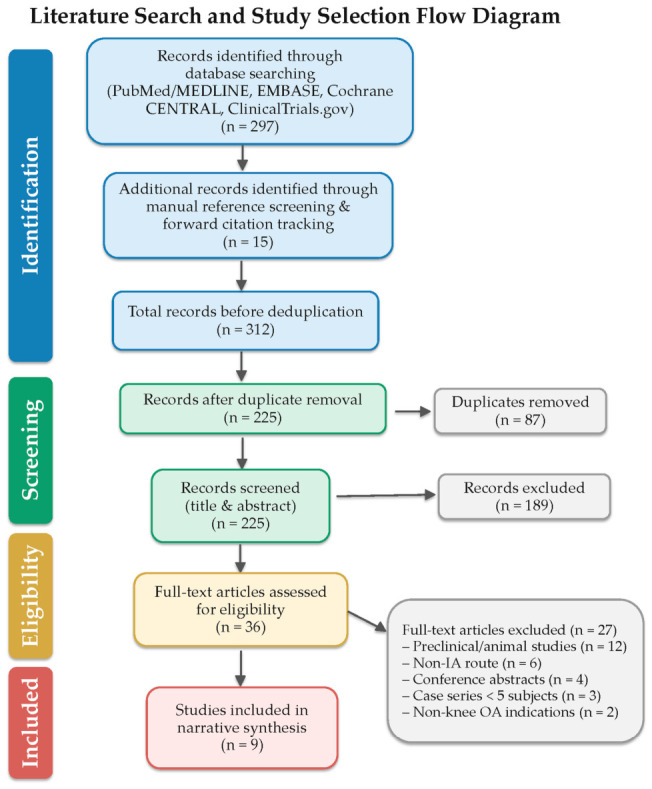
Literature Search and Study Selection Flow Diagram. A total of 312 records were identified through electronic database searches (PubMed/MEDLINE, EMBASE, Cochrane CENTRAL, and ClinicalTrials.gov; *n* = 297) and manual reference list screening and forward citation tracking (*n* = 15). After removal of 87 duplicate records, 225 unique citations underwent title and abstract screening, of which 189 were excluded for irrelevance to the target population, intervention, or study type. The remaining 36 full-text articles were assessed for eligibility, and 27 were subsequently excluded for the following reasons: purely preclinical or animal-based studies without human clinical data (*n* = 12), non-intra-articular route of administration (*n* = 6), conference abstracts without available peer-reviewed full text (*n* = 4), case reports or series with fewer than five subjects (*n* = 3), and non-knee OA indications without relevant subgroup data (*n* = 2). A final total of 9 studies met all predefined inclusion criteria and were incorporated into the narrative synthesis.

This narrative review employed a structured and transparent literature search approach, designed to enhance reporting transparency and reproducibility. Reporting quality was guided by the Scale for the Quality Assessment of Narrative Review Articles (SANRA) [22], which provides a structured framework specifically designed for the evaluation and reporting of narrative reviews. Although the format of the literature search and study selection flow diagram (Figure 1) superficially resembles diagrams commonly used in systematic reviews, such as those described in the PRISMA 2020 statement [23], this narrative review did not follow PRISMA methodology. Formal protocol registration in PROSPERO or equivalent registries was therefore not performed, and items specific to systematic reviews—including formal risk of bias assessment (RoB 2/ROBINS-I), GRADE certainty evaluation, and quantitative data pooling—were not applicable to this narrative synthesis.

A structured electronic literature search was conducted across PubMed/MEDLINE, EMBASE, the Cochrane Central Register of Controlled Trials (CENTRAL), and ClinicalTrials.gov, covering publications from January 2015 to December 2025, reflecting the period during which clinically relevant human studies on amniotic and chorionic membrane biologics for knee osteoarthritis have emerged. Searches were conducted using a combination of MeSH terms and free-text keywords related to amniotic and chorionic membrane biologics, knee osteoarthritis, and IA injection, guided by the PICO framework. Supplementary searches were performed using product-specific trade names, including “BioDRestore,” “Clarix Flo,” “AmnioFix,” and “HASA,” to capture commercially relevant literature that is not indexed under generic terminology. Reference lists of the identified articles were manually reviewed for additional eligible studies, and forward citation tracking was performed using Google Scholar and Scopus for key foundational publications [15,18,19]. ClinicalTrials.gov was searched to identify registered trials and to cross-reference their published outcomes with the corresponding peer-reviewed literature; unpublished or terminated trials lacking a corresponding peer-reviewed full-text publication were not incorporated into the primary evidence synthesis.

Studies were considered eligible if they enrolled adult patients with a clinical or radiographic diagnosis of knee OA and reported outcomes following the IA injection of any amniotic or chorionic membrane-derived biological product, including mdHACM, dHACM, ASA, HASA, cryopreserved amniotic membrane, or morselized amniotic/chorionic tissue formulations. The eligible outcome domains included patient-reported outcome measures (PROMs), such as KOOS, WOMAC, VAS, IKDC, Lysholm, and SANE scores, as well as safety, adverse event, and immunologic data. All prospective and retrospective clinical study designs involving human participants were considered, provided that full-text peer-reviewed manuscripts were available in English. Preclinical or animal-based studies, case reports or series involving fewer than five participants, conference abstracts, and studies evaluating non-IA routes of administration were excluded from the primary evidence synthesis. The threshold of five participants was adopted a priori as a pragmatic cutoff to exclude case reports and very small case series that are typically considered to provide a lower level of evidence and higher susceptibility to selection and reporting bias; this threshold is consistent with common practice in narrative and scoping reviews, although we acknowledge it was not derived from a formal statistical justification.

Retrieved citations were first deduplicated and screened at the title and abstract levels, followed by full-text reviews of potentially eligible records. Relevant data were extracted from the included studies. These included the study design, patient demographics, intervention and comparator details, follow-up duration, PROM scores, safety outcomes, and immunogenicity data, where available. The complete study selection process is summarized in the literature search and study selection flow diagram (Figure 1).

Given the heterogeneity in study designs, product formulations, patient populations, and outcome measures across the included studies, formal meta-analysis pooling was not performed. Rather, the findings were synthesized and organized around the themes of comparative clinical efficacy, safety and immunological profiles, and the identification of critical evidence gaps. The chronological evolution of the field was also considered to contextualize the progression from early cryopreserved formulations to contemporary dehydrated and micronized products.

## 3. Clinical Evidence Synthesis

### 3.1. Literature Search and Study Selection

An electronic database search yielded 312 records before deduplication. After removing 87 duplicate records, 225 unique citations underwent title and abstract screening. Of these, 189 were excluded as clearly irrelevant to the target population, intervention, or study type. The remaining 36 full-text articles were assessed for eligibility, and 27 were subsequently excluded for the following reasons: purely preclinical or animal-based studies (*n* = 12); non-IA route of administration (*n* = 6); conference abstracts without available peer-reviewed full text (*n* = 4); case reports or series with fewer than five participants (*n* = 3); and non-knee OA indications without relevant subgroup data (*n* = 2). Nine studies met all inclusion criteria and were included in the final narrative synthesis. The complete study selection process is illustrated in the literature search and study selection flow diagram (Figure 1).

### 3.2. Characteristics of Included Studies

The included studies were published between 2015 and 2025. They spanned six distinct amniotic/chorionic membrane product categories: ASA (*n* = 3 studies), morselized amniotic tissue/BioDRestore (*n* = 1), HASA (*n* = 1), cryopreserved amniotic membrane/suspension (CAM; *n* = 2), mdHACM (*n* = 1), and AM/UC particulate (*n* = 1). The study designs included RCTs (*n* = 4), prospective cohort or pilot studies (*n* = 3), and retrospective case series (*n* = 2). The sample sizes ranged from 6 to 200 patients, and the follow-up duration ranged from 24 weeks to 12 months. The characteristics of all the included studies are summarized in Table 1. Before interpreting the efficacy data across studies, it is important to distinguish between the two cryopreserved formulations referenced in this review. Cryopreserved amniotic suspension allograft (ASA; e.g., ReNu^®^) consists of particulate amniotic membrane tissue and amniotic fluid components suspended in a cryoprotective medium, designed for direct intra-articular injection. In contrast, CAM refers to intact or minimally processed amniotic membrane tissue preserved in sheet or particulate form. While both formulations retain bioactive constituents including growth factors and TIMPs, their cellular composition, bioactive factor concentrations, and intra-articular residence and release characteristics may differ substantially. Accordingly, efficacy data derived from ASA trials (e.g., Farr et al. [19], Gomoll et al. [20]) should not be extrapolated to CAM-based products without independent clinical validation.

### 3.3. Clinical Efficacy Outcomes

The clinical efficacy outcomes of the nine included studies are described below and cross-referenced with Table 1. The most robust comparative evidence was derived from a multicenter, single-blind RCT by Farr et al. [19], which enrolled 200 patients randomized to receive a single IA injection of ASA, HA, or saline. At the primary endpoint of 6 months, ASA demonstrated statistically significant superiority over both HA and saline across multiple KOOS domains, including Pain and Activities of Daily Living. The treatment failure rates at 3 months were markedly lower in the ASA group (13.2%) than in the HA group (approximately 68%), representing a clinically and statistically significant difference [19]. The 12-month follow-up data reported by Gomoll et al. [20] confirmed the durability of these effects, with mean KOOS Pain improvements of +14.3 (LOCF) and +17.7 (MMRM) points from baseline; both improvements exceeded the established minimum clinically important difference. A prespecified crossover analysis further demonstrated that patients who had failed HA or saline at 3 months and subsequently received ASA achieved functional improvements comparable to those observed in the original ASA group, suggesting that the therapeutic benefit of ASA is reproducible and may represent a viable salvage option for patients who do not respond to conventional IA therapies [20].

A double-blind RCT [21] presented a methodological advancement by directly comparing a morselized amniotic tissue product (BioDRestore) with triamcinolone acetonide in 81 patients with severe KL grade 3–4 knee OA. Although no statistically significant between-group differences in primary PRO measures were observed at 12 months, a clinically significant divergence in the trajectory of improvement was identified. The corticosteroid group demonstrated the expected pattern of early symptomatic relief, followed by gradual decay. In contrast, the BioDRestore group exhibited continued improvement in SANE, Lysholm, and KOOS measures between 6 weeks and 12 months. This pattern is consistent with a more sustained biological mechanism of action [21]. The inclusion of a population with severe OA in this trial extends the potential applicability of amniotic biologics beyond the mild-to-moderate OA spectrum typically studied.

The most substantive direct clinical evidence for mdHACM was provided in a retrospective case series comprising 82 patients (100 knees) treated with a single IA injection of 100 mg mdHACM [15]. Statistically significant improvements in the KOOS Pain and Function subscales were observed at 6 months, providing an initial human clinical signal for this formulation. However, the retrospective uncontrolled design precludes the definitive attribution of these improvements to the mdHACM intervention, as contributions from natural history, regression to the mean, and placebo effects cannot be excluded. The absence of KL grade stratification and the 6-month follow-up horizon further limit the interpretability and generalizability of these findings.

Uncontrolled prospective evidence from smaller cohort studies provides further support. Natali et al. [25] reported statistically significant and sustained improvements in VAS pain and IKDC scores 12 months after a single IA injection of 3 mL HASA in 25 patients with symptomatic knee OA. Similarly, Castellanos and Tighe [26] reported clinically meaningful improvements in the WOMAC and VAS scores at 24 weeks following a single IA injection of 50 mg of AM/UC particulates in 20 patients. The earliest human clinical evidence was derived from Vines et al. [18], who published the first prospective pilot study of IA cryopreserved amniotic suspension in patients with knee OA. Their findings demonstrated clinically meaningful symptomatic improvements without serious adverse events at 6 months [18]. This pivotal proof-of-concept provided a basis for subsequent controlled investigations. Similarly, a complementary retrospective CAM series reported improvements in patient-reported outcomes; no serious adverse events were reported [27]. Collectively, these findings established the foundational feasibility and safety framework on which the field has developed.

### 3.4. Safety, Immunological, Structural, and Economic Outcomes

A consistent and reassuring safety profile was observed across all the included studies. No serious adverse events attributable to amniotic or chorionic membrane biologics were reported. Additionally, no cases of joint infection, accelerated OA progression, systemic immune complications, or anaphylactic reactions were identified across the combined study populations. The most rigorous immunological monitoring was performed within the ASA RCT program; Gomoll et al. [20] conducted serial measurements of serum immunoglobulin levels and anti-HLA antibody titers for 12 months in a 200-patient cohort. No clinically relevant elevations in anti-HLA antibodies or abnormal immunoglobulin trends were detected. These observations provide strong evidence against a meaningful alloimmune response. This finding is clinically relevant, given the allogeneic nature of these products and the potential for subsequent joint arthroplasty in this patient population. Minor transient adverse events were infrequently reported. Specifically, Natali et al. [25] reported a 16% incidence of self-resolving events, including a brief burning sensation, mild joint pain, and transient synovitis-type flares, following HASA injection. In contrast, no comparable systematic safety monitoring was reported in the mdHACM series by Alden et al. [15].

Contrary to the consistent symptomatic and safety data, objective structural, biomarker, and economic outcomes were either absent or severely limited across all included studies. Radiographic assessments were included in the ASA RCT at 12 months but yielded no significant between-group differences in joint space width or radiographic OA progression [20]. Quantitative MRI-based structural outcome measures—T2 mapping, T1ρ relaxometry, and cartilage volume quantification—were entirely absent from all included human clinical studies. This critical evidence gap limited the assessment of potential disease-modifying effects beyond symptom-based endpoints. Similarly, none of the included studies reported synovial fluid or serum biomarker data beyond immunological safety parameters; data for pro-inflammatory cytokines, matrix metalloproteinases, or cartilage degradation markers were uniformly unavailable. From a health economic perspective, none of the included studies reported formal cost-effectiveness analyses, calculated quality-adjusted life years, or incremental cost-effectiveness ratios, despite the substantially higher acquisition cost of amniotic biological products than that of conventional IA comparators. This limitation represents a significant barrier to payer coverage decisions and widespread clinical adoption.

Finally, the literature search revealed no prospective comparative clinical studies evaluating amniotic membrane biologics for secondary indications, including MRI-confirmed IA tendon or ligament injuries, or post-traumatic OA as a distinct patient population. These indicate complete evidence gaps in human participants despite the biological plausibility supported by preclinical data [13]. Moreover, these limitations highlight the need for targeted clinical investigations in these underexplored populations.

## 4. Discussion

This narrative review synthesizes the available clinical evidence for IA amniotic and chorionic membrane-derived biologics in knee OA from nine studies published between 2015 and 2025. Our analyses reveal a biologically plausible and clinically promising product class, supported by an encouraging but still early stage and heterogeneous evidence base. Among the included formulations, ASA carries the most robust evidentiary support, with multicenter RCT data demonstrating statistically significant and clinically meaningful symptomatic improvements compared to HA and saline at both 6 and 12 months [19,20]. The safety and immunological profiles of amniotic and chorionic membrane products were consistently favorable across all studied formulations. However, direct prospective controlled evidence for mdHACM remains absent. Although the existing retrospective data for this formulation are encouraging, they are insufficient to support definitive conclusions regarding its efficacy or optimal clinical positioning. Thus, these observations should be interpreted in the context of the inherent methodological limitations of the included studies and substantial heterogeneity across product formulations, patient populations, and outcome measures. The limitations of existing IA therapies include transient corticosteroid effects with potential cartilage toxicity [6], inconsistent HA efficacy [7], and heterogeneous PRP outcomes [9]. Therefore, amniotic biologics may warrant further investigation. Specifically, the current evidentiary foundation must be appropriately strengthened through rigorous prospective trials.

The ASA RCT, comprising the reports by Farr et al. [19] and Gomoll et al. [20] from the same 200-patient multicenter cohort, provided the strongest available evidence for amniotic biologics as a class. ASA exhibited superiority over HA at 6 months, with treatment failure rates of 13.2% and approximately 68% in the ASA and HA groups, respectively. This indicates the need for reconsideration of the relative clinical utility of HA viscosupplementation in symptomatic knee OA, a therapy whose efficacy has been questioned in several systematic reviews and meta-analyses [7]. The durability of ASA benefits over 12 months, with KOOS Pain improvements exceeding the established MCID [20], further distinguishes its effect profile from that of corticosteroids, whose symptomatic benefits typically plateau or decay within 4–8 weeks and are associated with potential cartilage toxicity following repeated administration [6]. Additionally, a pre-specified crossover analysis suggested that ASA retains its therapeutic effect when administered as a second-line intervention following HA failure [24]. This finding has practical implications for treatment sequencing, although the open-label nature of this crossover limits the strength of inference. Nevertheless, the single-blind design of the primary RCT introduced the possibility of a differential placebo response, which is well-documented in IA injection trials and may account for a substantial proportion of the observed symptomatic improvement [28]. This should be considered when interpreting the magnitude of between-group differences reported in this study.

**Figure 2:**
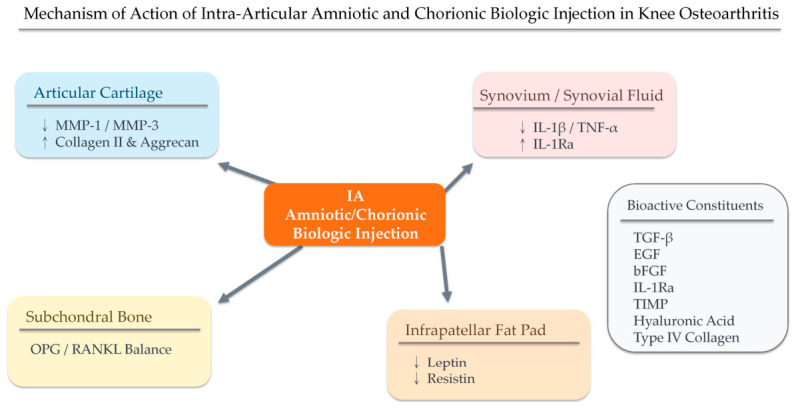
Proposed mechanism of action of intra-articular amniotic and chorionic mem brane biologics in the knee joint microenvironment. Amniotic and chorionic membrane-derived biologics exert coordinated effects across four tissue compartments: (1) Articular cartilage—suppression of matrix metalloproteinase (MMP)-1/3 activity and promotion of type II collagen and aggrecan synthesis; (2) Synovium and synovial fluid—reduction in pro-inflammatory cytokines (IL-1β, TNF-α), upregulation of IL-1 receptor antagonist (IL-1Ra), and stimulation of endogenous hyaluronic acid (HA) synthesis; (3) Subchondral bone—modulation of osteoprotegerin (OPG)/receptor activator of nuclear factor-κB ligand (RANKL) balance to attenuate pathological bone remodeling; and (4) Infrapatellar fat pad (IFP)—attenuation of adipokine-driven inflammation through reduction in leptin and resistin secretion. Key bioactive constituents include transforming growth factor-β (TGF-β), epidermal growth factor (EGF), basic fibroblast growth factor (bFGF), IL-1Ra, tissue inhibitors of metalloproteinases (TIMP)-1/2, hyaluronic acid, and type IV collagen. Arrows indicate the direction of regulatory effect: ↑, upregulation or increase; ↓, downregulation or decrease.

The double-blind RCT reported by Pill et al. [21] represents a methodological advancement. This study employed allocation concealment and blinding of both patients and outcome assessors to compare morselized amniotic tissue (BioDRestore) with triamcinolone acetonide in 81 patients with severe KL grade 3–4 knee OA. Although no statistically significant between-group differences were observed in the primary PRO measures at 12 months, the divergent trajectories of improvement between the groups—continued incremental gain in the amniotic group versus early peak and gradual decay in the corticosteroid group—are of potential clinical interest. This pattern is mechanistically plausible given that corticosteroids exert their effects primarily through rapid but transient suppression of synovial inflammation via glucocorticoid receptor-mediated transcriptional inhibition [29]. In contrast, amniotic membrane-derived biologics are hypothesized to modulate the IA microenvironment through sustained mechanisms, including TGF-β-mediated signaling, TIMP-mediated inhibition of matrix metalloproteinases, and growth factor support of chondrocyte homeostasis [11,13]. The proposed multi-compartment mechanisms of action of intra-articular amniotic and chorionic membrane biologics, spanning articular cartilage, synovium, subchondral bone, and the infrapatellar fat pad, are schematically illustrated in Figure 2. However, whether this trajectory divergence translates into clinically meaningful differences beyond 12 months remains unknown. Furthermore, the absence of a saline placebo arm in this study limits the ability to distinguish active treatment effects from placebo responses in either group. This design consideration should be addressed in future clinical trials. Nonetheless, the inclusion of a population with severe OA suggests that amniotic biologics may be worth investigating across the full OA severity spectrum rather than being restricted to mild-to-moderate disease.

The retrospective case series by Alden et al. [15] provided the most substantive direct evidence for mdHACM. This study reported significant improvements in KOOS Pain and functional subscales at 6 months following a single 100 mg IA injection in 100 knees. Although these findings provide preliminary clinical evidence supporting the feasibility of mdHACM as an IA therapy, the uncontrolled retrospective design substantially limits causal inference. Contributions from natural disease fluctuations, regression to the mean, concurrent treatments, and placebo effects cannot be excluded. In addition, the absence of KL grade stratification limits the characterization of the treated population. Furthermore, although ASA and mdHACM are both derived from human amnion and chorion tissue, their processing methodology and physical form substantially differ ASA is a cryopreserved liquid suspension, whereas mdHACM undergoes dehydration and micronization. These differences may meaningfully alter the bioavailability and IA residence time of the constituent bioactive molecules. Therefore, the clinical efficacy data for ASA cannot be assumed to be directly transferable to mdHACM. Product-specific prospective controlled data are required before mdHACM can be confidently positioned within the treatment landscape for knee OA.

The safety profile observed across all the included studies was reassuring and internally consistent. No serious adverse events attributable to any amniotic or chorionic membrane biologic were reported across the combined study population. Furthermore, rigorous immunological monitoring in the ASA RCT demonstrated no clinically relevant anti-HLA antibody formation or immunoglobulin abnormalities over 12 months in 200 patients [20]. This is particularly relevant given the allogeneic nature of these products and the theoretical concern regarding sensitization in patients who may subsequently undergo total knee arthroplasty. However, the completeness of the current safety characterization has major limitations. The maximum follow-up period across all the included studies was 12 months, which may be insufficient to detect delayed immunological sensitization or long-term effects on OA progression. In addition, systematic adverse event monitoring varied substantially across studies. Furthermore, none of the included studies evaluated the potential impact of prior amniotic biological exposure on subsequent arthroplasty outcomes, a clinically relevant question that warrants prospective investigation.

Beyond symptomatic outcomes, the current evidence base is deficient in several domains that are increasingly recognized as important for the comprehensive assessment of IA biologics. With respect to structural outcomes, quantitative MRI-based measures—T2 mapping, T1ρ relaxometry, and cartilage volume quantification—were entirely absent from all included human clinical studies. Consequently, the potential disease-modifying effects remain unaddressed at the human level. Preclinical data reported by Willett et al. [14] and Lin et al. [17] suggest that mdHACM attenuates cartilage degradation in animal models of OA, providing a biological rationale for investigating structural endpoints in future human trials. However, the translation of preclinical structural findings to clinical imaging outcomes cannot be assumed. The incorporation of validated quantitative MRI techniques as secondary endpoints in future trials would provide objective evidence to complement the symptom-based PRO data that constitute the current human evidence base [30]. With respect to mechanistic biomarkers, no studies have reported synovial fluid or serum data beyond immunological safety parameters, leaving the biological basis of the observed clinical improvements largely uncharacterized. Measurement of proinflammatory cytokines, matrix metalloproteinases, and cartilage degradation markers in future trials could provide mechanistic insights and potentially identify predictive biomarkers for treatment response. However, the clinical utility of such biomarkers in guiding IA biological therapy remains to be established [31]. With respect to health economics, the absence of formal cost-effectiveness analyses across all the included studies represents a meaningful gap, particularly given the substantially higher acquisition costs of amniotic biological products than those of conventional IA comparators. Although EQ-5D-5L utility data were collected in some trials [19,21], none of the included studies calculated the QALYs or ICERs. This information is likely to be required by payers and health technology assessment bodies before broader coverage decisions can be made.

Several additional gaps in evidence merit acknowledgment. First, no prospective comparative clinical studies evaluating amniotic membrane biologics specifically for post-traumatic OA or for IA tendon and ligament pathology have been identified despite the biological plausibility supported by preclinical data [17]. Second, all included clinical studies enrolled patients with primary degenerative knee OA without specific stratification for PTOA. As such, the generalizability of the current findings to this distinct pathophysiological subgroup remains unclear. Finally, the optimal dose, injection frequency, and timing of amniotic biological administration relative to the OA stage remain undefined; existing data do not permit evidence-based recommendations on these parameters.

**Figure 3:**
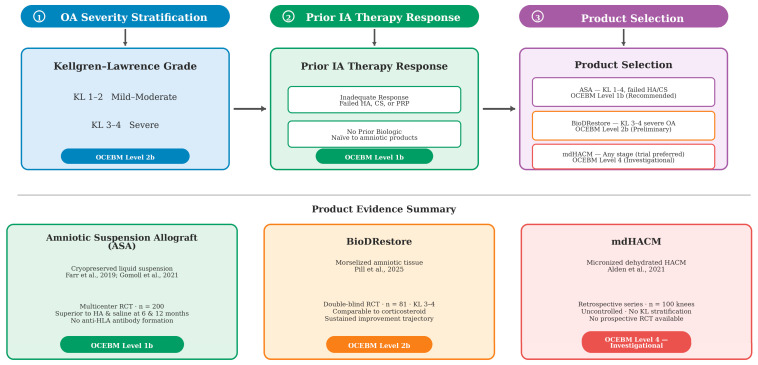
Clinical decision framework for intra-articular amniotic and chorionic membrane biologics in knee osteoarthritis. This conceptual framework summarizes the current evidence hierarchy across three sequential clinical considerations: (1) osteoarthritis severity stratification according to Kellgren–Lawrence grade, (2) response to prior intra-articular therapy, and (3) product selection. Amniotic suspension allograft (ASA) has the strongest evidentiary support, based on the multicenter randomized controlled trial reported by Farr et al. [19] and its 12-month follow-up reported by Gomoll et al. [20]. BioDRestore is supported by preliminary randomized evidence in patients with severe knee osteoarthritis reported by Pill et al. [21]. Micronized dehydrated human amnion/chorion membrane (mdHACM) is currently supported primarily by the retrospective case series reported by Alden et al. [15] and should therefore be considered investigational pending prospective controlled studies. The framework is intended as a structured summary of the available evidence and does not constitute a formal clinical practice guideline. Clinical decisions should incorporate individual patient characteristics, prior treatment response, local product availability, and shared decision-making. Abbreviations: ASA, amniotic suspension allograft; BioDRestore, morselized amniotic tissue; mdHACM, micronized dehydrated human amnion/chorion membrane; HA, hyaluronic acid; CS, corticosteroid; IA, intra-articular; KL, Kellgren–Lawrence; OA, osteoarthritis; OCEBM, Oxford Centre for Evidence-Based Medicine; RCT, randomized controlled trial.

Based on the evidence reviewed, we propose a conceptual framework for the clinical consideration of IA amniotic and chorionic membrane biologics in knee OA (Figure 3). This framework is intended as a structured summary of the current evidence hierarchy to assist clinicians in contextualizing available options and does not constitute a formal clinical practice guideline. It is organized into three clinical considerations: OA severity, prior IA therapy response, and product selection, each annotated with its corresponding level of evidence. The framework acknowledges that ASA occupies the strongest evidentiary position [19,20]; BioDRestore provides preliminary data on severe OA [21]; and mdHACM use outside of clinical trial settings may reasonably be considered investigational pending prospective controlled data [15]. Clinicians using these products in routine practice should engage patients in shared decision-making that transparently communicates the current evidentiary limitations.

Despite the encouraging clinical signals summarized above, a critical appraisal of the included evidence reveals several methodological limitations that warrant explicit acknowledgment. First, considerable product heterogeneity exists across the reviewed studies, with amniotic and chorionic membrane preparations varying substantially in tissue source, processing method (dehydrated, cryopreserved, micronized, or liquid suspension), cellular viability, and bioactive constituent content. As differences in processing may meaningfully alter the concentration of key mediators—including TGF-β, EGF, IL-1Ra, and hyaluronic acid—direct cross-study comparisons of efficacy must be interpreted with caution. Second, significant heterogeneity in outcome measurement was observed, with studies employing diverse PROMs (VAS, NRS, WOMAC, KOOS, IKDC) without standardized assessment timepoints, limiting cross-study comparability and precluding pooled quantitative analysis. Future studies would benefit from adopting the OMERACT core outcome set for OA clinical trials as a standardized reporting framework [32]. Third, the available evidence is predominantly limited to follow-up periods of ≤12 months. Given the chronic and progressive nature of knee OA, the durability of any symptomatic or structural benefit beyond this horizon remains unknown—a critical gap when positioning amniotic biologics relative to established IA therapies for which longer-term data are available.

This narrative review has several inherent limitations that should be considered when interpreting its findings. First, as a narrative review, this synthesis is subject to the methodological constraints of this format, including the absence of formal effect size pooling and the potential for author interpretation bias; these limitations were mitigated through a structured four-database search strategy and SANRA-guided reporting [22]. Second, publication bias represents a significant concern in this emerging field, as studies reporting positive outcomes are more likely to be indexed, potentially leading to an overestimation of therapeutic effects; the small number of eligible studies (*n* = 9) and the absence of registered trial protocols for several included studies further limit our ability to assess its extent. Third, restriction to English-language publications may have excluded relevant studies in Chinese, Korean, Japanese, and European languages, potentially resulting in an incomplete representation of the global evidence base. Fourth, the biological and methodological heterogeneity of the included studies—as discussed above—limits the generalizability of the synthesized findings, with variations in product formulation, injection protocol, patient selection, and outcome instruments precluding definitive conclusions regarding optimal dosing, timing, or patient selection. Fifth, the paucity of follow-up data beyond 12 months represents a critical gap for establishing treatment durability, disease-modifying potential, and the long-term safety profile of repeated intra-articular amniotic biologic injections. Notwithstanding these limitations, this narrative review represents the most comprehensive synthesis to date of the available clinical evidence for intra-articular amniotic and chorionic membrane biologics in knee OA and provides a structured foundation for the design of future randomized controlled trials in this field.

## 5. Conclusions

IA amniotic and chorionic membrane-derived biologics represent a clinically promising therapeutic class for knee OA, with a consistently favorable safety profile across all the studied formulations. Among the available products, ASA is supported by the highest level of evidence, demonstrating durable symptomatic superiority over HA and saline over 12 months in multicenter RCTs. However, direct prospective controlled evidence for mdHACM remains absent. In addition, fundamental gaps in structural, biomarker, and cost-effectiveness data persist across the field. A well-designed, multicenter, double-blind RCT incorporating structural, biomarker, and health economic endpoints—with follow-up durations extending beyond 12 months—is required to completely characterize the clinical role of mdHACM and to inform evidence-based patient selection and shared decision-making.

## Figures and Tables

**Table 1:** Summary of included studies evaluating intra-articular amniotic and/or chorionic membrane biologics for knee osteoarthritis (2015–2025).

Study (Year) [Ref.]	Design	*n*	Product Type and Formulation	Comparator	Primary Outcome Measure	Follow-Up	Key Finding
Pill et al. (2025) [21]	Double-blind RCT	81	BioDRestore (morselized amniotic tissue, single injection)	Triamcinolone acetonide	SANE, Lysholm, KOOS	12 months	Comparable short-term efficacy to corticosteroid; continued functional improvement trajectory through 12 months in amniotic group
Gomoll et al. (2021) [20]	Single-blind RCT	200	ASA (cryopreserved suspension, single injection)	HA, Saline	KOOS Pain	12 months	Sustained KOOS Pain improvement exceeding MCID; no clinically relevant anti-HLA antibody formation
Farr et al. (2019) [19]	Single-blind RCT	200	ASA (cryopreserved suspension, single injection)	HA, Saline	KOOS Pain, Function	6 months	Statistically significant superiority over HA and saline; treatment failure rate 13.2% (ASA) vs. ~68% (HA)
Gomoll et al. (2023) [24]	Single-blind RCT (crossover)	200	ASA (cryopreserved suspension, rescue injection)	HA/Saline → ASA rescue	KOOS Pain	12 months	Crossover ASA injection achieved comparable functional improvement to initial ASA group
Alden et al. (2021) [15]	Retrospective case series	82 (100 knees)	mdHACM (100 mg, single injection)	None	KOOS Pain, Function	6 months	Statistically significant improvements in KOOS Pain and Function at 6 months; no serious adverse events
Natali et al. (2022) [25]	Prospective pilot	25	HASA (3 mL, single injection)	None	VAS, IKDC	12 months	Significant and sustained VAS and IKDC improvement; 16% incidence of mild, self-resolving adverse events
Vines et al. (2016) [18]	Prospective pilot	6	Cryopreserved amniotic suspension (single injection)	None	PROs, Safety	12 months	Clinically meaningful symptomatic improvement; no serious adverse events; established early feasibility
Castellanos & Tighe (2019) [26]	Prospective pilot	20	AM/UC particulate (50 mg, single injection)	None	WOMAC, VAS	24 weeks	Clinically meaningful improvements in WOMAC and VAS at 24 weeks
Timmons et al. (2020) [27]	Retrospective case series	40	Cryopreserved amniotic membrane (single injection)	None	PROs, Safety	6 months	Improvements in patient-reported outcomes; no serious adverse events reported

Abbreviations: ASA, amniotic suspension allograft; HASA, human amniotic suspension allograft; mdHACM, micronized dehydrated human amnion/chorion membrane; AM/UC, amniotic membrane/umbilical cord; RCT, randomized controlled trial; HA, hyaluronic acid; KOOS, Knee Injury and Osteoarthritis Outcome Score; VAS, Visual Analogue Scale; IKDC, International Knee Documentation Committee score; WOMAC, Western Ontario and McMaster Universities Osteoarthritis Index; SANE, Single Assessment Numeric Evaluation; MCID, minimal clinically important difference; PROs, patient-reported outcomes; KL, Kellgren-Lawrence.

## Data Availability

All the data were within the article.

## Abbreviations

The following abbreviations are used in this manuscript:

OA	Osteoarthritis
IA	Intra-articular
HA	Hyaluronic acid
PRP	Platelet-rich plasma
TGF-β	Transforming growth factor-beta
TIMPs	Tissue inhibitors of metalloproteinases
mdHACM	Micronized dehydrated human amnion/chorion membrane
PTOA	Post-traumatic OA
ASA	Amniotic suspension allograft
RCT	Randomized controlled trial
PROMs	Patient-reported outcome measures
